# Antibacterial Composite Materials Based on the Combination of Polyhydroxyalkanoates With Selenium and Strontium Co-substituted Hydroxyapatite for Bone Regeneration

**DOI:** 10.3389/fbioe.2021.647007

**Published:** 2021-04-07

**Authors:** Elena Marcello, Muhammad Maqbool, Rinat Nigmatullin, Mark Cresswell, Philip R. Jackson, Pooja Basnett, Jonathan C. Knowles, Aldo R. Boccaccini, Ipsita Roy

**Affiliations:** ^1^School of Life Sciences, College of Liberal Arts and Sciences, University of Westminster, London, United Kingdom; ^2^Institute of Biomaterials, Department of Materials Science and Engineering, University of Erlangen-Nuremberg, Erlangen, Germany; ^3^Lucideon Ltd., Stoke-on-Trent, United Kingdom; ^4^CAM Bioceramics B.V., Leiden, Netherlands; ^5^Bristol Composites Institute (ACCIS), University of Bristol, Bristol, United Kingdom; ^6^Division of Biomaterials and Tissue Engineering, Faculty of Medical Sciences, University College London Eastman Dental Institute, London, United Kingdom; ^7^Department of Nanobiomedical Science and BK21 Plus NBM, Global Research Center for Regenerative Medicine, Dankook University, Cheonan, South Korea; ^8^The Discoveries Centre for Regenerative and Precision Medicine, University College London, London, United Kingdom; ^9^Department of Materials Science and Engineering, Faculty of Engineering, The University of Sheffield, Sheffield, United Kingdom

**Keywords:** antibacterial, composites, polyhydroxyalkanoates, selenium, strontium, hydroxyapatite

## Abstract

Due to the threat posed by the rapid growth in the resistance of microbial species to antibiotics, there is an urgent need to develop novel materials for biomedical applications capable of providing antibacterial properties without the use of such drugs. Bone healing represents one of the applications with the highest risk of postoperative infections, with potential serious complications in case of bacterial contaminations. Therefore, tissue engineering approaches aiming at the regeneration of bone tissue should be based on the use of materials possessing antibacterial properties alongside with biological and functional characteristics. In this study, we investigated the combination of polyhydroxyalkanoates (PHAs) with a novel antimicrobial hydroxyapatite (HA) containing selenium and strontium. Strontium was chosen for its well-known osteoinductive properties, while selenium is an emerging element investigated for its multi-functional activity as an antimicrobial and anticancer agent. Successful incorporation of such ions in the HA structure was obtained. Antibacterial activity against *Staphylococcus aureus* 6538P and *Escherichia coli* 8739 was confirmed for co-substituted HA in the powder form. Polymer-matrix composites based on two types of PHAs, P(3HB) and P(3HO-co-3HD-co-3HDD), were prepared by the incorporation of the developed antibacterial HA. An in-depth characterization of the composite materials was conducted to evaluate the effect of the filler on the physicochemical, thermal, and mechanical properties of the films. *In vitro* antibacterial testing showed that the composite samples induce a high reduction of the number of *S. aureus* 6538P and *E. coli* 8739 bacterial cells cultured on the surface of the materials. The films are also capable of releasing active ions which inhibited the growth of both Gram-positive and Gram-negative bacteria.

## Introduction

Bone tissue has a dynamic structure with high capacity of regeneration. However, its healing capability is linked with the proximity of the bone segments and, in the case of large-size defects, mechanical fixation on its own is not able to induce bone healing, requiring the use of additional material. These critical-size defects can be caused by a variety of scenarios including trauma, tumor resection, or bacterial infections (De Witte et al., 2018; Hasan et al., 2018). Moreover, external intervention is also required in the presence of fractures that have failed to heal completely (i.e., non-unions) (Stewart, 2019). In clinical practice, autologous osseous material is still considered the gold standard, thanks to its inherent osteogenic, osteoconductive, and osteoinductive properties. However, the restricted availability of this material combined with possible donor-site morbidity limits autograft applications, especially for the treatment of large bone defects (Fernandez de Grado et al., 2018; Zeng et al., 2018). Allografts and xenografts represent possible alternatives but are associated with the risk of an immunogenic response and disease transmission correlated with a reduction in the osteoinductive properties and a lack of viable cells (Baldwin et al., 2019).

In light of the limitations of the current transplanted osseous grafts, the use of tissue engineering has emerged as one of the main alternatives to restore or regenerate the damaged tissue through the combination of cells with a scaffold able to provide a suitable environment for cell anchorage and proliferation (Fernandez de Grado et al., 2018; Hasan et al., 2018). Among the characteristics of an ideal material for bone regeneration, increasing interest has been placed on the antibacterial properties. Infections represent a major complication in the orthopedic field, as bone repair is associated with a high risk of infections, especially in the presence of open fractures. If infection occurs, the tissue healing process might be impaired and, if not treated, can result in chronic infection, leading to bone necrosis and spreading to adjacent soft tissues (Thomas and Puleo, 2011). The main strategies applied in clinic to prevent the rise of or fight possible infections are based on antibiotics. For prevention, systemic delivery of antibiotics is employed which can be ineffective due to low drug concentration at the infected site and can lead to systemic toxicity (Mouriño and Boccaccini, 2010). Alternatively, grafts loaded with antibiotics are used, allowing a controlled release of the therapeutics at the targeted site and lowering possible side effects (Johnson and García, 2015). However, the increasing rise of antibiotic-resistant species due to the overuse or misuse of antibiotics has become a primary concern worldwide, posing a serious threat to global health and economy. Such a scenario requires the investigation of new materials for bone regeneration able to provide appropriate antibacterial features without the use of antibiotics to inhibit bacterial growth and reduce bacterial adhesion.

Composite materials are one of the best candidates for scaffolds for bone regeneration due to their ability to closely mimic the intrinsically heterogeneous composite architecture of such tissue, composed of cells, an organic phase (the extracellular matrix containing mainly collagen fibrils), an inorganic phase [mainly hydroxyapatite (HA)], and water (Chocholata et al., 2019; Qu et al., 2019). The most investigated combination in scaffold design involves the use of polymers to provide elasticity and biodegradability, mimicking the role of the extracellular matrix, and ceramics to increase the mechanical strength and improve the interactions between the tissue and the final constructs (Kashirina et al., 2019).

Hydroxyapatite Ca_10_(PO_4_)_6_(OH)_2_ is a versatile member of the calcium phosphate family with a stoichiometric composition similar to natural bone, Ca/P ratio 1.67. It is the main ceramic material used in the fabrication of composite scaffolds for bone regeneration thanks to its exceptional biocompatibility and bioactivity, in addition to osteoconductive properties (Ratnayake et al., 2017; Kaur et al., 2019). Moreover, the compressive strength of HA is similar to that of native bone, but the material possesses a brittle nature with low fracture toughness, limiting its use in load-bearing applications and requiring its combination with polymeric materials (Rezwan et al., 2006; Chocholata et al., 2019). Natural apatite has superior physical and biological properties as compared to synthetic HA because of a considerable hetero-ionic exchange (Eliaz and Metoki, 2017). For this reason, increasing research is being conducted to improve the physiochemical, biological, and mechanical properties of HA through the substitution of various metallic ions in the crystal structure of HA (Ratnayake et al., 2017). Among the ions investigated for substitution, selenium, an essential trace element, has attracted attention only in the last decade. Selenium plays a crucial role in the human body as an antioxidant through its incorporation in selenoproteins (proteins containing selenocysteine residues), specifically the glutathione peroxidase family, which are essential for protection against oxidative stress (Rodríguez-Valencia et al., 2013). Its deficiency has been associated with two diseases, the Kashin–Beck syndrome, which is characterized by cartilage and long bone degeneration, and the Keshan illness, which affects the heart in children (Moreno-Reyes et al., 2001; Rodríguez-Valencia et al., 2013). Moreover, selenium-containing biomaterials have shown an anticancer activity, inducing apoptosis of bone cancer cells *in vitro* and inhibiting the growth of bone tumors *in vivo* (Yanhua et al., 2016; He et al., 2019). Finally, recently, selenium has been shown to possess antibacterial activity against a range of bacteria (Kolmas et al., 2014; Huang et al., 2019). Strontium is one of the most studied elements for bone regeneration. Thanks to its osteoconductive and osteoprogenic properties, this ion has been incorporated into a range of materials for bone regeneration, such us titanium implant coatings, bone cements, and synthetic HA (Wong et al., 2004; Gritsch et al., 2019). Strontium has been shown to elicit a bone remodeling effect through the increase of osteoblast activity inducing bone formation and the reduction of osteoblast proliferation and activity, therefore reducing bone reabsorption (Nielsen, 2004; Ratnayake et al., 2017).

As regards the polymeric matrix for composite materials for bone regeneration, polyhydroxyalkanoates (PHAs) have been considered promising candidates due to their biocompatibility, biodegradability, easy processability, and versatile and tuneable physical and mechanical properties (Rai et al., 2011; Możejko-Ciesielska and Kiewisz, 2016). PHAs are bioderived polyesters, naturally produced by bacteria as intracellular compounds. The production from natural and renewable resources makes PHAs a good alternative to petrol-derived plastics. Moreover, PHAs have shown to possess less acidic degradation products than other synthetic polyesters such as PLA, PLGA, and PGA, limiting the possibility of developing a late or chronic inflammation (Bonartsev et al., 2019). Compared to other natural-derived materials (e.g., collagen and chitosan), PHAs show better mechanical features and higher thermal stability making them suitable for a wider range of processing techniques (Puppi et al., 2019). Depending on the bacteria and the carbon source utilized in the fermentation process, two types of PHAs can be obtained, short chain length PHAs (scl-PHAs), characterized by up to five carbon atoms in their monomeric unit, and medium chain length PHAs (mcl-PHAs), with more than six carbon atoms in their monomeric unit. These two classes of PHAs usually exhibit distinctive properties. Scl-PHAs are stiff and brittle materials, with a high melting temperature (160–180°C) and glass transition close to 0°C (Możejko-Ciesielska and Kiewisz, 2016; Puppi et al., 2019). On the contrary, mcl-PHAs usually show a higher elongation at break and a lower elastic modulus, melting temperature (40–60°C), and glass transition (−50 to 25°C) compared to scl-PHAs (Rai et al., 2011; Możejko-Ciesielska and Kiewisz, 2016; Basnett et al., 2018). Scl-PHAs are the main polyesters of the PHAs family investigated for bone regeneration due to their high crystallinity and mechanical properties (i.e., Young’s modulus and ultimate tensile strength), close to those of native bone (Karageorgiou and Kaplan, 2005). However, the *in vivo* application of these materials is limited by their brittle nature (Obat et al., 2013). Therefore, recently mcl-PHAs have also been investigated as an alternative material for bone regeneration in non-load bearing applications (Ansari et al., 2016).

In this work, we investigated the development of novel antibacterial materials based on the combination of PHAs with a novel co-substituted HA to be used as starting materials for the development of scaffolds for bone regeneration. In the first part of this research, we reported the synthesis and physiochemical characterization of novel selenium and strontium co-substituted HA (Se-Sr-HA) prepared by the co-precipitation method. *In vitro* characterization of the antibacterial properties of Se-Sr-HA was conducted to validate its utilization for the development of antimicrobial composites. The combination of the novel co-substituted HA with PHAs was then investigated. Two PHAs were selected as the bulk materials: P(3HB) as an scl-PHA and P(3HO-co-3HD-co-3HDD) as an mcl-PHA. The polyesters were combined with the novel co-substituted HA using three loading compositions, and the effect of the incorporation of the filler on the physical, thermal, mechanical, and antibacterial properties of the composite films was investigated.

## Materials and Methods

### Bacterial Strains and Culture Conditions

*Pseudomonas mendocina* CH50 and *Bacillus subtilis* OK2 were obtained from the University of Westminster culture collection. The strains were cultured at 30°C at 200 r/min for 16 h in a shaking incubator and stored as a glycerol stock at −80°C. For the antibacterial characterization, *Staphylococcus aureus* 6538P and *Escherichia coli* 8739 were bought from the American Type Culture Collection (ATCC). They were cultured in sterile nutrient broth at 37°C and 200 r/min for 16 h in a shaking incubator and stored as a glycerol stock at −80°C.

### Chemicals

All the chemicals were purchased from Sigma Aldrich Ltd., United Kingdom and VWR international, United Kingdom.

### Synthesis of Selenium-Strontium-Hydroxyapatite

Selenium-strontium-HA was synthesized by wet precipitation method, using diammonium hydrogen phosphate (NH_4_)_2_HPO_4_, calcium nitrate tetrahydrate Ca(NO_3_)_2_4H_2_O, sodium selenite Na_2_SeO_3_⋅5H_2_O, and strontium nitrate Sr(NO_3_)_2_⋅6H_2_O as precursors. Two solutions coded A and B were prepared for this reaction. Solution A was prepared my dissolving Ca(NO_3_)_2_⋅4H_2_O (0.668 M) and Sr(NO_3_)_2_⋅6H_2_O (0.167 M) in 250 mL deionized water and adjusted to pH 11 using 1 M NH_4_OH. Solution B was prepared by dissolving (NH_4_)_2_HPO_4_ (0.4 M) and Na_2_SeO_3_⋅5H_2_O (0.1 M) in 250 mL deionized water and adjusted to pH 9.5 using 1 M NH_4_OH. The level of reactants used was calculated in order to keep the (Ca+Sr):(P+Se) ratio constant at 1.667 and Sr:(Sr+Ca) and Se:(Se+P) molar ratio constant at 0.2. The aimed chemical composition of the produced Se-Sr-HA was Ca_10__–__x_Sr_x_(PO_4_)_6__–__*y*_(SeO_3_)_*y*_(OH)_2__–__*y*_, where x = 2 and y = 1.2 (Ravi et al., 2012; Pajor et al., 2018). Solution B was added dropwise to solution A. After mixing of the solutions, the pH value was maintained at 10.75 by adding NH_4_OH, and the final solution was stirred for 16 h at 400 r/min followed by the aging of precipitates for 24 h. The obtained precipitates were washed with de-ionized water and then centrifuged (three times). The samples were dried at 80°C in an oven and sintered at 900°C for 6 h (at the heating rate of 5°C/min). Non-substituted HA was prepeared with the same method without the addition of Na_2_SeO_3_⋅H_2_O and Sr(NO_3_)_2_⋅6H_2_O.

### Physicochemical Characterization of Se-Sr-HA

#### X-Ray Diffraction (XRD) Analysis

X-ray diffraction (XRD) analysis was conducted on a Bruker D8 Advance using Bragg-Brentano parafocusing geometry. Data were collected for the 2θ range from 10° to 70° (step size of 0.01° and count time 1 s per step). Collected data were qualitatively examined using the Bruker diffract+ and EVA search-match software for compounds identification (using the ICDD database).

#### X-Ray Fluorescence Analysis (XRF)

For compositional analysis by XRF, the samples were prepared and reported in accordance with ISO standard 12677. The substituted HA samples were ignited at 1200°C for 1 h to examine the loss on ignition. Then approximately, 1.5 g powdered sample was mixed up with 7.5 g of Li_2_B_4_O_7_ (lithium tetraborate) and heated in a platinum crucible at 1025°C for 20 min followed by 1200°C for 5 min. Then the glass melt was cast into a glass disc to get a glassy bead which was analyzed by using Axios’ Panalytical X-Ray Fluorescence (XRF) spectrometer.

### Production of Polyhydroxyalkanoates

P(3HO-co-3HD-co-3HDD) was produced by *P. mendocina* CH50 using 20 g/L of coconut oil as previously described by Basnett et al. (2018). P(3HB) was produced by the controlled fermentation of *B. subtilis* OK2 using 35 g/L of glucose as the carbon source as described by Lukasiewicz et al. (2018). Both fermentation processes were carried out in 15 L bioreactors, with 10 L working volume (Applikon Biotechnology, Tewkesbury, United Kingdom). The polymers were extracted using a two-stage method Soxhlet extraction (Lukasiewicz et al., 2018).

### Composite Films Preparation and Characterization

Polyhydroxyalkanoates/Se-Sr-HA composite films were obtained by solvent casting technique. P(3HO-co-3HD-co-3HDD) or P(3HB) was first dissolved in chloroform (5% w/v) at room temperature and stirred for 24 h. The desired amount of filler (i.e., 10, 20, and 30 wt% of Se-Sr-HA) was added in the polymer solution, and the mixture was then sonicated in an ultrasonic water bath (XUBA3 Ultrasonic Bath, Grant Instruments) for 15 min. The obtained suspension was then poured in petri-dishes and left to evaporate at room temperature. Samples were kept for 5 weeks to allow complete crystallization of the samples before conducting the following characterizations (Lukasiewicz et al., 2018; Lizarraga Valderrama et al., 2020).

#### Attenuated Total Reflectance Fourier Transform Infrared (ATR-FTIR) Spectroscopy

Attenuated total reflectance Fourier transform infrared (ATR-FTIR) was carried out to investigate the functional groups of the composite films. The analyses were performed in a spectral range of 4000 to 400 cm^–1^ with a resolution of 4 cm^–1^ using PerkinElmer FT-IR spectrometer Spectrum Two (PerkinElmer Inc., United States).

#### Differential Scanning Calorimetry (DSC)

Thermal analysis of the films was conducted using differential scanning calorimetry (DSC) 214 Polyma (Netzsch, Germany) equipped with Intracooler IC70 cooling system. Approximately 5 mg of the sample was heated at a heating rate of 20°C per minute from −70 to 130°C for P(3HO-co-3HD-co-3HDD)-based samples and from −70 and 200°C for P(3HB)-based samples (Lukasiewicz et al., 2018; Lizarraga Valderrama et al., 2020). The data were analyzed using the Proteus 7.0 Analysis Software (Netzsch, Germany). The enthalpy of fusion (Δ*H*m) of the composite samples was normalized $\left(\Delta \,H_{\text{m}}^{n}\right)$ to take into account the weight fraction of the filler (w_*f*_):

$$\Delta \,H_{\text{m}}^{n}=\frac{\Delta \,H\,\text{m}}{\left(1-w_{f}\right)}$$

For P(3HB)-based samples, the percentage crystallinity of the materials (*X*_*c*_%) was calculated according to the following formula:

$$X_{c}\%=\frac{\Delta \,H\,\text{m}}{\Delta \,H^{\circ }}*100$$

Where Δ*H*m is the enthalpy of fusion of the material and Δ*H*° is the enthalpy of fusion for the material with 100% crystallinity, which for P(3HB) is 146 J/g (Ho et al., 2014). In case of composite films, Δ*H*m was replaced with Δ*H_n_*_m_.

#### Scanning Electron Microscopy (SEM) and Energy-Dispersive X-Ray (EDX) Spectroscopy

The surface topography of the composite samples was analyzed through scanning electron microscopy (SEM) using a beam of 5 keV at 10 cm working distance (JEOL 5610LV-SEM). Energy-dispersive X-ray (EDX) analysis was used to perform a qualitative elemental analysis of the materials developed with a beam of 20 keV at 10 cm working distance. The data were analyzed using the software IncaEDX Energy System. For both analyses, all the samples were coated with gold for 2 min using an EMITECH-K550 gold sputtering device. The analyses were carried out at the Eastman Dental Hospital, University College London.

#### Mechanical Analysis

Tensile testing was performed on the composite films using Instron 5940 testing system equipped with 500 N load cell. Solvent casted films of 5 mm width and 35 mm length were used for the analysis, and six specimens were used for each sample. A deformation rate of 10 mm/min was applied for mcl-PHAs, while 5 mm/min was used for scl-PHAs following the ASTM D882–10 “Standard Test Method for Tensile Properties of Thin Plastic Sheeting” (Lukasiewicz et al., 2018; Lizarraga Valderrama et al., 2020). The data were acquired and analyzed using a BlueHill 3 software.

### Antibacterial Characterization

#### Minimal Inhibitory Concentration (MIC)

The antibacterial activity of Se-Sr-HA in powder form was investigated using the ISO 20776 against *S. aureus* 6538P and *E. coli* 8739. A range of concentration of the material in powder form (5–100 mg/mL) prepared in Mueller Hinton broth was mixed with a microbial suspension adjusted to achieve a final concentration of 5 × 10^5^ CFU/mL and incubated at 37°C for 24 h at 100 r/min in 96 multi-well plates (final volume 100 μL). After the incubation time, the OD_600_ of the wells was measured to determine the concentration of the Se-Sr-HA able to inhibit the growth [minimal inhibitory concentration (MIC)] of each bacterial strain.

#### Direct Contact Test—ISO 22196

The antibacterial properties of the composite films were evaluated following the ISO 22196 against *S. aureus* 6538P and *E. coli* 8739. Samples of 6 mm in diameter were sterilized by UV light for 15 min, placed onto agar plates, inoculated with 10 μL of a bacterial culture adjusted to a concentration of 3–5 × 10^5^ CFU/mL and incubated in static conditions at 37°C for 24 h. After the incubation time, the bacteria were recovered from the sample using a PBS solution and the number of viable cells was determined through the colony-forming unit method using the drop plate technique (Herigstad et al., 2001). P(3HO-co-3HD-co-3HDD) or P(3HB) neat samples were used as controls. The antibacterial activity (R%) was expressed as the % reduction of the number of cells, which was calculated using the formula:

$$R\left(\%\right)=\frac{U-T}{U}*100$$

Where *U* is the number of viable bacteria normalized to the surface area of the sample, in CFU/cm^2^, recovered from the control samples, and *T* is the number of viable bacteria, in CFU/cm^2^, recovered from samples containing Se-Sr-HA.

#### Antibacterial Ion Release Studies

Antibacterial ion release studies were performed on composite films against *S. aureus* 6538P or *E. coli* 8739, following an adapted method described by Cao et al. (2017). Samples of 6 mm in diameter were incubated in 1 mL of Mueller Hinton broth at 37°C for 1, 3, 6, and 24 h. After each time point, the media with the eluted ions were collected and replaced with fresh media. The obtained eluates were incubated with a microbial suspension adjusted to 3–5 × 10^5^ CFU/mL and incubated for 24 h at 37°C in 96 multi-well plates (final volume 100 μL). The control consists of Mueller Hinton broth incubated for the same time period with P(3HO-co-3HD-co-3HDD) or P(3HB) neat samples. The antibacterial activity was calculated as the % reduction of the OD at 600 nm compared to the control using the following formula:

$$\%OD_{600}reduction=\frac{\left(O\,D_{c}-O\,D_{s}\right)}{O\,D_{c}}*100$$

OD_*c*_ is the optical density at 600 nm of the control samples and OD_*s*_ is the optical density at 600 nm of the sample.

### Statistical Analysis

All measurements were made in triplicate unless otherwise specified, and data are presented as mean values ± standard deviation. Statistical analysis was performed using GraphPad Prism 7 software by one-way analysis of variance (ANOVA) with Tukey *post hoc* test to determine statistically significant differences between two or more groups. The differences were considered statistically significant when the *p*-values resulted lower than 0.05 [*p*-value < 0.05 (^∗^), *p*-value < 0.01 (^∗∗^), *p*-value < 0.001 (^∗∗∗^), and *p*-value < 0.0001 (^****^)].

## Results

### Se-Sr-HA Characterization

#### XRD Analysis

**FIGURE 1:**
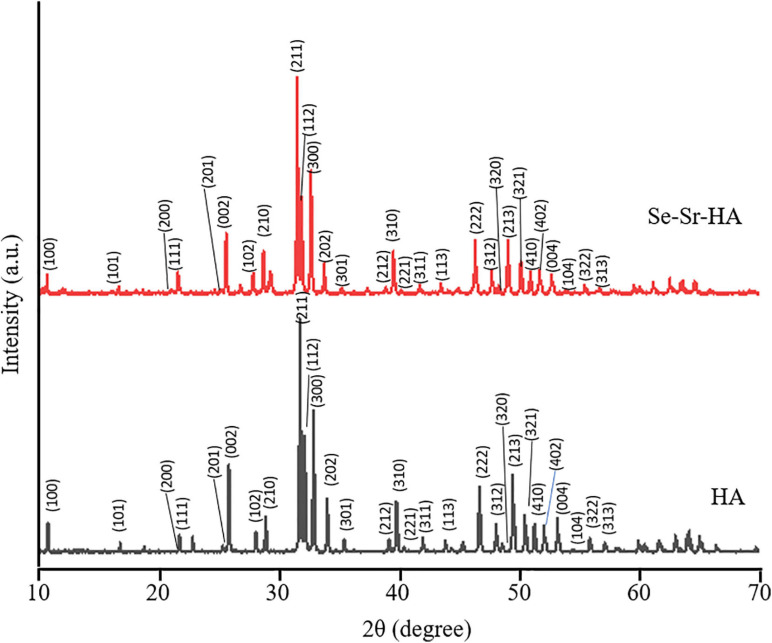
X-ray diffractograms of non-substituted hydroxyapatite (HA) and selenium ion substituted hydroxyapatite (Se-Sr-HA) synthesized by the wet precipitation method and sintered at 900°C. The characteristic diffraction peaks for hexagonal HA could be detected in both HA and Se-Sr-HA samples. Reflections originating from hexagonal HA phase are labeled with their corresponding Miller indices.

Figure 1 shows powder X-ray diffractograms of HA and Se-Sr-HA. Both diffractograms were characteristic of highly crystalline materials that matched the standard diffractogram of HA (JCPDS. 09-432). The characteristic diffraction peaks for hexagonal HA could be detected in both HA and Se-Sr-HA samples. The co-substituted HA showed broadening of the peaks and slight reduction in the peak intensities, indicating the incorporation of ions in the HA lattice structure. Moreover, a minor shift in the diffraction peaks of Se-Sr-HA toward lower angle values (2θ) as compared to reference HA peaks was also detected, a further confirmation of ion doping (Ozeki et al., 2014).

Measurements of the lattice parameters of HA and Se-Sr-HA were performed by Rietveld refinement, and are reported in Table 1. The substitution of selenium and strontium ions caused the increase in a-axis and c-axis lattice parameters of Se-Sr-HA as compared to reference HA. An expansion of the unit cell volume of Se-Sr-HA as compared to pure HA was also detected.

**TABLE 1:** Lattice parameters of HA and Se-Sr-HA.

Sample	*α-*axis (A°)	*c*-axis (A°)	Unit cell volume (A°)^3^
**HA**	9.443	6.865	528.03
**Se-Sr-HA**	9.506	6.961	536.91

#### XRF Analysis

Table 2 shows the mol.% of the elements present in HA and Se-Sr-HA calculated through semi-qualitative XRF analysis. For the co-substituted HA, the presence of strontium and selenium could be detected along with calcium and phosphorus, confirming the substitution of both the selenium and strontium ions into the lattice structure of HA. Se-Sr-HA samples showed a Ca/P molar ratio 1.53, slightly lower than the stoichiometric Ca/P molar ratio in pure HA which is equal to 1.667. The slight difference might be the semi-quantitative mode of analysis.

**TABLE 2:** The measured elemental composition of HA and Se-Sr-HA.

Samples	Calcium	Phosphorus	Selenium	Strontium	Ca+Sr/P+Se ratio	
	(mol.%)	(mol.%)	(mol.%)	(mol.%)	Theoretically calculated	XRF
HA	1.09	0.65	0	0	1.67	1.67
Se-Sr-HA	0.82	0.55	0.0441	0.0897	1.67	1.53

### Antibacterial Characterization of Se-Sr-HA

**FIGURE 2:**
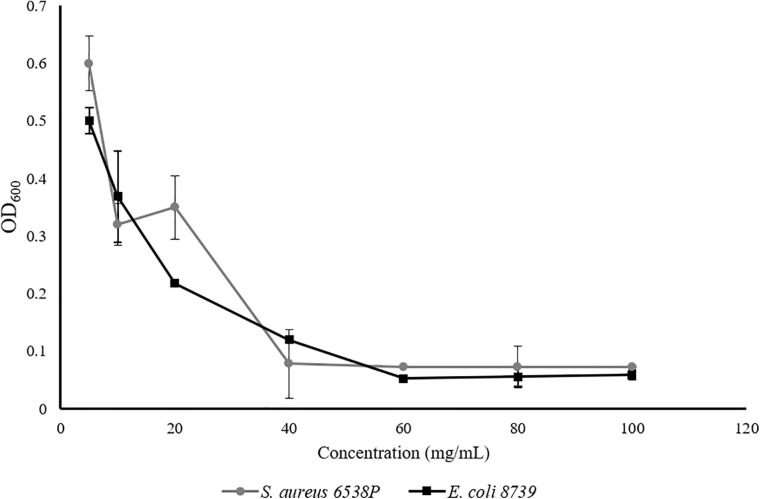
MIC analysis of Se-Sr-HA powders against *S. aureus 6538P* (in grey) and *E.coli 8739* (in black). For both bacteria, the variations of the OD_600_ with an increase in the concentration of Se-Sr-HA are plotted.

To evaluate whether the novel Se-Sr-HA possessed any antibacterial properties, a preliminary *in vitro* characterization was conducted following the ISO 20776 to determine the MIC (i.e., the lowest concentration of an antimicrobial agent that inhibits the visible growth of the bacteria) using the broth dilution test against *S. aureus* 6538P and *E. coli* 8739. Figure 2 shows the variation of the optical density at 600 nm as a function of the Se-Sr-HA concentration against the two bacterial species. In both curves, a similar trend could be detected characterized by the inhibition of bacterial growth with increasing concentration of Se-Sr-HA. The MIC against *S. aureus* 6538P was 40 mg/mL, while the value for *E. coli* 8739 was 60 mg/mL.

### Physicochemical Characterization of 2D Antibacterial Composite Scaffolds

**FIGURE 3:**
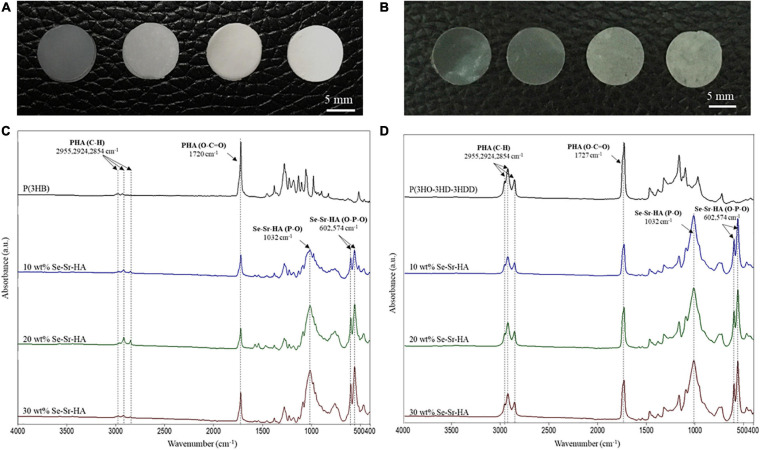
Optical images of P(3HB) **(A)** and P(3HO-co-3HD-co-3HDD) **(B)** composite films. In each image, the samples are in the following order from left to right: neat film, 10 wt% Se-Sr-HA, 20 wt% Se-Sr-HA, and 30 wt% Se-Sr-HA composite films. ATR-FT-IR spectra of P(3HB) **(C)** and P(3HO-co-3HD-co-3HDD) **(D)** composite films. In each spectrum, neat samples are in black, 10 wt% Se-Sr-HA in blue, 20 wt% Se-Sr-HA in green, and 30 wt% Se-Sr-HA in red.

To obtain 2D antibacterial composite scaffolds, three different loading compositions of Se-Sr-HA were investigated, 10, 20 and 30 wt% using P(3HB) or P(3HO-co-3HD-co-3HDD) as the matrix material. Figure 3 shows the appearance of composite films of different compositions and corresponding FTIR spectra. From macroscopic observation the presence of HA in the films could be detected, as the material looked white and opaque.

#### ATR-FTIR Analysis

Qualitative characterization of P(3HB) and P(3HO-co-3HD-co-3HDD) composites was conducted by ATR-FTIR and the spectra are shown in Figures 3C,D, respectively. In all the spectra, the characteristic peak of PHAs related to the stretching of the carbonyl group (1720–1740 cm^–1^) in the ester bond was identified. For scl-based composites, this peak is at 1720 cm^–1^, while for mcl-based films at 1727 cm^–1^. The mcl-PHA-based composites also showed more prominent peaks around 2900 cm^–1^ [i.e., stretching of carbon–hydrogen bond of methyl and methylene group (CH_3_, CH_2_)] compared to the scl-PHAs (Randriamahefa et al., 2003; Kann et al., 2014). The spectra of all the composite films showed new bands at 1072–1032, 601, 571, and 474 cm^–1^, which were assigned to the vibrations of the phosphate group, PO_4_^–3^, present in the HA. In particular, the area between 1072 and 1032 cm^–1^ is related to the stretching mode of the phosphorus oxygen (P–O) bond in the phosphate group, while the area below 600 cm^–1^ is due to the bending mode of the O–P–O bond in the phosphate group (Koutsopoulos, 2002; Li et al., 2007).

#### SEM and EDX Analyses

**FIGURE 4:**
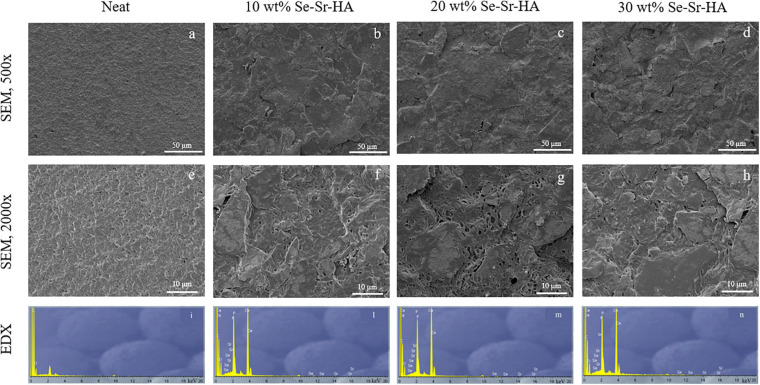
SEM image at 500× **(a–d)** and 2000× **(e–h)** and EDX spectra **(i–n)** of P(3HB) based composite films. The samples are in the following order from left to right: neat film, 10 wt% Se-Sr-HA, 20 wt% Se-Sr-HA, and 30 wt% Se-Sr-HA composite films.

**FIGURE 5:**
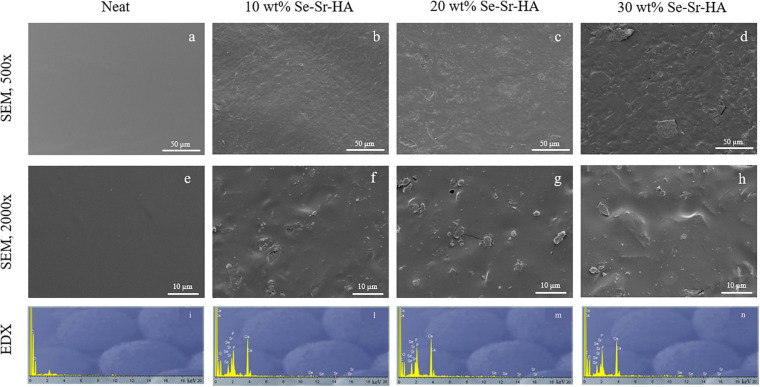
SEM image at 500× **(a–d)** and 2000× **(e–h)** and EDX spectra **(i–n)** of P(3HO-co-3HD-co-3HDD) based composite films. The samples are in the following order from left to right: neat film, 10 wt% Se-Sr-HA, 20 wt% Se-Sr-HA, and 30 wt% Se-Sr-HA composite films.

Scanning electron microscopy and EDX analyses of the surface of P(3HB) and P(3HO-co-3HD-co-3HDD) composite films are reported in Figures 4, 5, respectively. For both types of PHAs, the surface of the composite films appeared less uniform than that of the neat samples due to the presence of HA particles. EDX analyses confirmed the presence of Se-Sr-HA particles in the materials. The spectra of the neat samples showed only the presence of peaks assigned to carbon and oxygen, while for all the composite films, the presence of the other four elements (i.e., calcium, phosphorus, selenium, and strontium) associated with Se-Sr-HA was detected.

### Thermal Characterization

The effect of the incorporation of Se-Sr-HA on the thermal properties of the composite films was evaluated by DSC analysis (Table 3). For both P(3HO-co-3HD-co-3HDD) and P(3HB) composite samples, the loading of HA did not induce statistically significant differences in the melting temperature and the glass transition values of the composite films as compared to the neat samples. Interestingly, none of the P(3HB)-based films showed the presence of a glass transition phase, including the neat samples. Such result indicates the transformation of the amorphous fraction of the polymer into a rigid amorphous phase due to vitrification during storage of the films at room temperature for 5 weeks (Lukasiewicz et al., 2018). The loading of the antibacterial HA into both types of polymeric matrices led to a significant reduction of the enthalpy of fusion (normalized to the content of the polymer) for both types of PHAs considered. For P(3HB)-based samples, the sample with 30 wt% of Se-Sr-HA showed a statistically significant reduction in the enthalpy of fusion and consequently the percentage crystallinity of the composite samples as compared to the neat samples

**TABLE 3:** Thermal properties of P(3HB) and P(3HO-co-3HD-co-3HDD) composite samples.

Sample		T_*m*_ (°C)	T_*g*_ (°C)	ΔH^*n*^_*m*_ (J/g)	*X*_*c*_ (%)
PHA	Wt% of Se-Sr-HA				
P(3HB)	0 (neat)	169 ± 2	n.d.	82 ± 4	56 ± 4
	10	169 ± 1	n.d.	83 ± 4	57 ± 3
	20	170 ± 1	n.d.	78.5 ± 4	54 ± 3
	30	169 ± 1.5	n.d.	61 ± 4.5	42 ± 3
P(3HO-3HO-3HDD)	0 (neat)	53 ± 5	−44 ± 2	24.5 ± 1	–
	10	52 ± 0.5	−40.5 ± 2	21.5 ± 1.5	–
	20	52.5 ± 0.5	−42 ± 2	19 ± 1.5	–
	30	52 ± 0.5	−42 ± 0.5	18 ± 2	–

*T_*m*_ is the melting peak, T_*g*_ is the glass transition, ΔH^*n*^_*m*_ is the enthalpy of fusion normalized to the mass fraction of the polymer, and *X*_*c*_ is the % polymer crystallinity.*

(*p*-value < 0.01). Moreover, the sample with 30 wt% of filler showed a statistically significant reduction in the values of enthalpy of fusion as compared to both samples with 20 wt% (*p*-value < 0.05) and 10 wt% (*p*-value < 0.01) of Se-Sr-HA. For P(3HO-co-3HD-co-3HDD) films, the samples with the highest contents of filler (i.e., 20 and 30 wt%) showed statistically lower values of enthalpy of fusion as compared to the neat films (*p*-value < 0.05 for 20 wt% and *p*-value < 0.01 for 30 wt%). However, no statistically significant differences in the enthalpy of fusion were detected between the composite films (i.e., 10 wt% vs 20 wt% vs 30wt%). For mcl-PHA-based materials, it was not possible to calculate the value of crystallinity from the enthalpy of fusion as the reference enthalpy of fusion (i.e., the enthalpy of fusion for a 100% crystalline polymer) is not known (Suchitra, 2004). Since the enthalpy of fusion is directly proportional to the degree of crystallinity, the decrease in the enthalpy of fusion of the composite indicated that the presence of the filler affected P(3HO-co-3HD-co-3HDD) crystallization.

### Mechanical Characterization

Tensile testing of the composite films was conducted to assess the influence of the incorporation of Se-Sr-HA on the mechanical properties of the materials, and the results are reported in Table 4. Overall, for both types of PHAs, the addition of HA leads to an increase in the elastic modulus and a decrease of the elongation at break of the composite samples as compared to the neat ones. For P(3HB) samples, the film containing 20 wt% of Se-Sr-HA showed the highest increase in the Young’s modulus, which was almost double that of the neat samples (*p*-value < 0.01). Also, the ultimate tensile strength increased by 1.5 times as compared with the neat films (*p*-value < 0.001). In terms of elongation at break, all the composite films showed a three to seven times decrease in the average values, without statistically significant difference between the three different compositions. As regards the mcl-PHA-based composites, Se-Sr-HA filler induced an increase in the elastic modulus by two to three times for all the compositions tested as compared to neat P(3HO-co-3HD-co-3HDD) samples, but no statistically significant differences were detected between the samples with different Se-Sr-HA content. In terms of the ultimate tensile strength, the samples with 30 wt% of filler showed a significant decrease in the average values compared to the neat films (*p*-value < 0.5), while for the other two filler contents (i.e., 10 and 20 wt%), no variation could be detected. Finally, a significant reduction of the elongation at break was detected for all the composite samples, with the highest average reduction of almost 2.5 times observed for the films with 30 wt% of Se-Sr-HA.

**TABLE 4:** Mechanical properties of P(3HB) and P(3HO-co-3HD-co-3HDD) composite films and corresponding neat polymers (*n* = 6).

Sample		Young’s modulus (MPa)	Ultimate tensile strength (MPa)	Elongation at break (%)
PHA	Wt% of Se-Sr-HA			
P(3HB)	0 (neat)	900 ± 120	20 ± 1.5	16 ± 8
	10	1100 ± 100	23.5 ± 1.0	5 ± 2
	20	1700 ± 200	30 ± 3	2.5 ± 1.0
	30	900 ± 200	24 ± 2	3 ± 1
P(3HO-3HO-3HDD)	0 (neat)	5 ± 2	6.5 ± 1.0	414 ± 32
	10	14 ± 4	7.0 ± 1.5	299 ± 44
	20	14 ± 6	6.0 ± 1.5	284 ± 31
	30	16 ± 2	4 ± 1	173 ± 32

### Antibacterial Characterization

The antibacterial properties of the composite films developed were investigated using two tests, a direct contact test (i.e., ISO 22196) and an indirect one (i.e., antibacterial ion release). The former is a standard well-established procedure used for the quantification of the antimicrobial properties of the surfaces of materials (Molling et al., 2014). The latter one was chosen to evaluate whether the materials produced release ions possessing antimicrobial activity. The 2D composite films developed are expected to exhibit an antibacterial activity by two mechanisms: contact activity when bacteria adhere to the material surface containing HA with selenite ions incorporated in its structure and due to an action of solubilized ions released in the surrounding environment. The two selected tests enable analysis of the contribution of the two mechanisms independently of each other.

#### Direct Contact Test—ISO 22196

**FIGURE 6:**
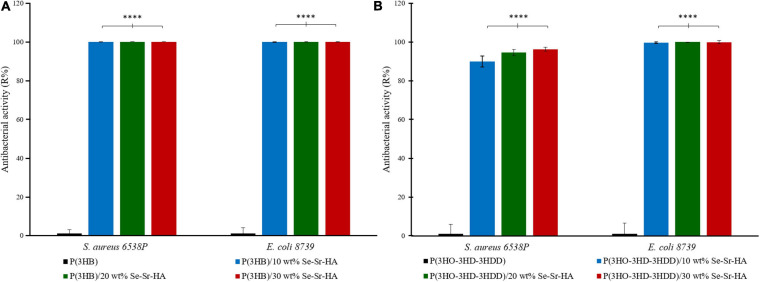
Antibacterial activity (R%) (ISO 22196) of P(3HB) **(A)** and P(3HO-co-3HD-co-3HDD) **(B)** composite films against *S. aureus* 6538P and *E. coli* 8739. In each figure, neat films are in black, composite films with 10 wt% of Se-Sr-HA in blue, 20 wt% in green, and 30 wt% red. The control consists of bacteria cultured on neat films (inducing no reduction in the bacterial cell number, having an average R% of zero). ^****^p-value < 0.0001 indicates statistically significant difference between the composite films and the respective neat films.

The ISO 22196 was performed to investigate the antimicrobial activity of the composite films against *S. aureus* 6538P and *E. coli* 8739, seeded directly on the surface of the materials. P(3HB) and P(3HO-co-3HD-co-3HDD) composite samples showed to be active against both bacterial species at all the three loading contents investigated, inducing a reduction of the bacterial cell number as compared to the respective neat samples (Figure 6). For the scl-PHA-based composites, all the films showed a high efficacy, causing a 100% killing of *S. aureus* 6538P and *E. coli* 8739 for all the compositions tested. The P(3HO-co-3HD-co-3HDD) composite films showed a bactericidal effect against *S. aureus* 6538P, increasing with the increase in the HA content. The samples with the lowest contents of filler (i.e., 10 and 20 wt% of Se-Sr-HA) induced a 90 and 94% average reduction of the number of bacterial cells, respectively, while the highest efficacy was obtained with the highest content of the filler (i.e., 30 wt% of Se-Sr-HA), causing an average 96% reduction of bacterial cells. The mcl-PHA-based composites showed a higher effect against *E. coli* 8739, as all the compositions investigated led to at least a 99.5% reduction of the bacterial cell count.

#### Antibacterial Ion Release Studies

**FIGURE 7:**
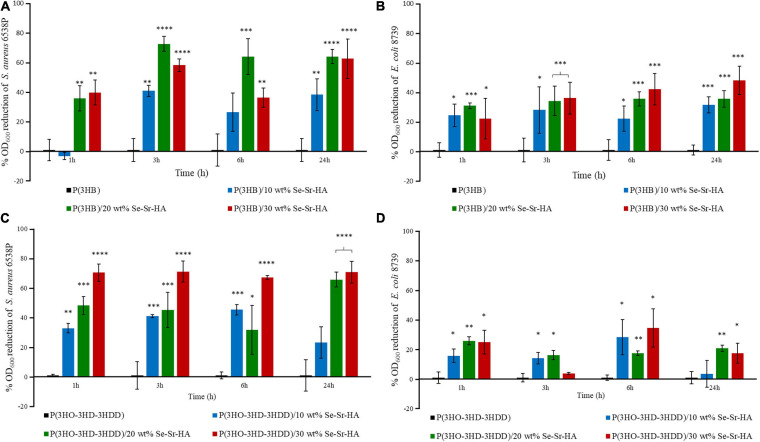
Indirect antibacterial ion release study of the eluates from P(3HB) **(A,B)** and P(3HO-3HD-3HDD) **(C,D)** composite films *S. aureus* 6538P **(A,C)** and *E. coli* 8739 **(B,D)**. In each figure, neat films are in black, composite films with 10 wt% of Se-Sr-HA in blue, 20 wt% in green, and 30 wt% in red. The control is bacteria cultured with the eluates obtained from the neat films (inducing no antibacterial effect, having an average %OD_600_ reduction of zero).^∗^*p*-value < 0.05, ^∗∗^*p*-value < 0.01, ^∗∗∗^*p*-value < 0.001, and ^****^p-value < 0.0001 indicate statistically significant difference between the composite samples and the respective neat films.

The antibacterial release study was performed against *S. aureus* 6538P and *E. coli* 8739 to investigate the capability of the developed 2D antibacterial composite films to release ions with antibacterial properties. In this indirect method, the bacterial cells were with the eluates obtained by incubating the polymeric samples in Mueller Hinton broth. The antibacterial activity was calculated as the reduction in the OD of the bacterial suspension in contact with the eluates obtained from the composite samples as compared to bacteria cultured with the eluates from the neat films. Overall, P(3HB) and P(3HO-co-3HD-co-3HDD) composite samples showed activity against both bacterial species, as shown in Figure 7. In the case of *S. aureus* 6538P, both materials showed at least a 30% average reduction of the OD for all the time points evaluated. Moreover, the composite with the highest content of HA (i.e., 20 and 30 wt%) showed a higher antibacterial activity than that of samples with 10 wt% of filler at all the time points evaluated. Finally, both materials induced a reduction of the OD of *E. coli* 8739 cells, even though the efficacy was lower as compared to *S. aureus* 6538P.

## Discussion

The use of HA doped with metal ions in bone regeneration has attracted great interest in recent years thanks to the possibility of enhancing its mechanical and biological properties. In this work, a novel co-substituted HA was developed by wet precipitation with sodium selenite and strontium nitrate as the sources of selenium and strontium, respectively. In the literature, HA containing either strontium or selenium has been developed (Uskokovic et al., 2017; Zarins et al., 2019), but to our knowledge to date, no study regarding the simultaneous co-substitution of such ions has been published. Selenium was chosen to introduce antibacterial properties in the material. Only a few studies have been conducted on the antibacterial properties of selenium and the mechanism behind its efficacy has not been elucidated yet. The main hypothesis formulated is the induction of oxidative stress through the production of reactive oxygen species and the depletion of thiol groups, inducing membrane and cell wall damage as well as permanent changes in cell components leading to bacterial death (Kolmas et al., 2014; Huang et al., 2019). However, the use of selenium has also been linked with cytotoxic effects (Wang et al., 2012; Uskokovic et al., 2017). For this reason, in this work, its co-substitution with strontium was investigated to off-set its toxicity. Strontium ions were chosen as they have been shown to introduce osteoinductive and osteogenic properties, favoring bone remodeling (Nielsen, 2004; Ratnayake et al., 2017). Structural characterization through XRD and XRF confirmed the co-substitution of both ions in the crystalline lattice. From the literature, it is known that strontium ions partially substitute calcium ions through cationic substitutions, while anionic substitution occurs between selenite and phosphate groups (Ratnayake et al., 2017; Uskokovic et al., 2017). The introduction of such ions induced a change in lattice parameters and an increase in the unit cell volume. Sr^2+^ might have played a significant role in the expansion of the unit cell volume of Se-Sr-HA due to its larger ionic radius as compared to Ca^2+^, 118 and 99 pm, respectively (Gritsch et al., 2019; Zarins et al., 2019). Nevertheless, Se-Sr-HA still maintained the crystalline structure of HA. In particular, the XRD results showed that selenite ions were substituted into the HA crystal lattice in the absence of secondary phase. Moreover, the XRD and XRF data confirmed the incorporation of selenium into the lattice structure of HA. If these ions were only adsorbed on the crystal surface, the content of selenium would have been negligible (as any soluble selenium salt would be removed during the washing step). The selenium and strontium content obtained in Se-Sr-HA samples was significantly lower than the anticipated values based on the stoichiometric ratio of components used in the synthesis (Table 2). Such a difference could be associated with the semi-quantitative mode of analysis used for XFR in this study. Moreover, the lower substitution of strontium ions at calcium sites could be related to the larger ionic radii of Sr^2+^(118 pm) compared to Ca^2+^ (99 pm). Selenite and phosphate ions have similar ionic radii, but a different geometric arrangement, flat trigonal pyramid structure for SeO_3_^2–^ compared to tetrahedral structure for PO_4_^3–^ ions, which could be the reason for the lower substitution (Pajor et al., 2018). Consequently, the added ions were partially substituted in the HA lattice, while the excess selenium and strontium ions remained in solution were removed during the washing steps of the wet precipitation synthesis.

A preliminary *in vitro* characterization of the materials confirmed that the introduction of selenium conferred antibacterial properties to the HA. The Se-Sr-HA showed to be active against both *S. aureus* 6538P and *E. coli* 8739, inducing inhibition of the microbial cell growth. The composition of the Se-Sr-HA used in this work [i.e., produced considering Sr:(Sr+Ca) and Se:(Se+P) molar ratios of 0.2] was selected based on their antibacterial properties. Other compositions of Se-Sr-HA were prepared by varying the Se/(Se+P) and Sr/(Sr+Ca) ratios (i.e., 0.01, 0.03, 0.05, and 0.1) and they exhibited a lower antibacterial activity compared to the one used in this study (i.e., 0.2) against both bacterial species (data not shown). Limited research has been conducted in the literature to investigate the antibacterial properties of selenium-doped HA, and the few studies carried out vary in terms of content of selenium, form of HA, and test method utilized, making it difficult to compare the results obtained. Nevertheless, overall the materials produced have shown activity against both Gram-positive and Gram-negative bacteria, as shown in this study. In the study by Uskokovic et al., substituted HAs with four concentrations of selenium (0.1, 1.2, 1.9, and 3 wt%) in the form of powders showed activity against *E. coli*, *S. aureus*, *Salmonella enteritidis*, and *Pseudomonas aeruginosa*, inducing a zone of inhibition at all the concentrations investigated. Rodríguez-Valencia et al. produced Se-HA with 0.6 at% of selenium. The ceramic produced was used as a coating for titanium disc samples and showed activity against *P. aeruginosa* 19582 and *S. aureus* 6538P, by inducing a reduction in biofilm formation (Rodríguez-Valencia et al., 2013). Finally, a co-substituted HA with selenium (3.6 wt%) and manganese (0.6 wt%) was produced by Kolmas et al. The antimicrobial properties of the material were investigated by the incubation of the ceramic in the form of pellets with a microbial suspension of *S. aureus* 25293 and *E. coli* 2592 for 24 h. The material induced reduction in the cell count of *S. aureus* 25293, while it showed no activity against *E. coli* 2592 (Kolmas et al., 2015). The difference in the form of HA used, pellet discs, compared to the powder used in this study and in the work of Uskokovic et al. might be the reason for the discrepancy in the results obtained against *E. coli*, as the former might have led to a lower release of antimicrobial ions compared to the free powder form. Nevertheless, our study and published works are in agreement that Se-HA is less efficient against *E. coli* than *S. aureus*.

The Se-Sr-HA produced was combined with two types of PHAs, P(3HB) and P(3HO-co-3HD-co-3HDD) to produce antibacterial composite materials for their potential use as the starting materials for the fabrication of scaffolds for bone regeneration. The two polymers were produced by bacterial fermentation following optimized protocols previously developed by our group (Basnett et al., 2018; Lukasiewicz et al., 2018). These two polyesters were chosen as representative of scl and mcl-PHAs, respectively. As mentioned in Section “Introduction,” P(3HB) has been explored in the literature as a material for bone tissue regeneration thanks to its mechanical properties, in the range of those of native bone (Karageorgiou and Kaplan, 2005; Misra et al., 2008). Little investigation has been conducted on the use of mcl-PHAs for bone regeneration. Ansari et al. (2016) developed a porous composite scaffold through the combination of P(3HHx-co-3HO) with non-substituted HA. The elastomeric nature of these polymers makes them attractive suitable candidates as materials for non-load bearing applications. In this work, we studied the combination of the two PHAs with Se-Sr-HA to produce novel composite materials with osteinductive and antibacterial properties, without the use of antibiotics.

Qualitative chemical analysis through FT-IR and EDX confirmed the incorporation of the Se-Sr-HA inside the polymer matrix. Analysis of the surface morphology evidenced differences between the P(3HB)-based and P(3HO-co-3HD-co-3HDD)-based samples. Mcl-PHA films obtained by solvent evaporation are usually characterized by a smooth surface, while scl-PHA films usually show a rougher surface with protrusions and pores (Rai et al., 2011; Anbukarasu et al., 2015). This distinctive feature can be attributed to the higher degree of crystallization and a more rapid crystallization rate of scl-PHAs compared to mcl-PHAs (Kai et al., 2003; Li et al., 2016). The two types of composite films with Se-Sr-HA showed the same distinctive characteristics. For P(3HB) composites, the presence of a more porous surface led to a higher exposure of the HA compared to the P(3HO-co-3HD-co-3HDD) ones. Wang et al. reported a similar behavior between the surface of P(3HB) and P(3HB-co-3HHx) films loaded with HA. A higher amount of the filling could be detected on the surface of the former composites, which was attributed to the higher crystallinity of the bulk material (Wang et al., 2008).

The loading of Se-Sr-HA in the two types of PHA-based composites induced a decrease in the enthalpy of fusion (and consequently the crystallinity) of the materials with high content of filler compared to the neat samples. These results indicate that the introduction of high quantities of filler reduced the mobility of the chains, hindering the material crystallization (Misra et al., 2008). Such behavior can be associated with the micro-size dimension of the filler utilized in this study. In the literature, hindering of the crystallization has been shown when combining PHAs with micro-size HA (Bergmann and Owen, 2003; Ansari et al., 2016), while a nucleating effect was detected in the presence of nano-sized HA particles (Misra et al., 2008; Noohom et al., 2009; Sadat-Shojai et al., 2013). The increase in crystallinity has been associated with the better interface between the filler and the polymeric matrix created by the higher dispersion of nano-sized HA compared with micro-size particles (Misra et al., 2008; Sadat-Shojai et al., 2013).

One of the main reasons for the use of composites is to create a final material that has a higher strength and modulus than the polymeric component and at the same time better toughness and processability than the inorganic filler. For both types of PHAs, the introduction of the HA led to an improvement in the strength and stiffness of the material. Simultaneously, an embrittlement of the constructs was evidenced through the reduction of the elongation at break. In particular, in the case of P(3HB), a composite loading percentage of 20 wt% was found to be optimal to obtain the highest increment of Young’s modulus (1.7 GPa) and tensile strength (30 MPa) compared with the neat P(3HB) films (0.9 GPa and 19 MPa, respectively). A higher content of filler (i.e., 30 wt%) induced a detrimental effect on the mechanical properties of the composite films with a decrease in both the elastic modulus and the tensile strength as compared to the samples with 20 wt% of Se-Sr-HA. Such behavior can be correlated to the decrease in the crystallinity of the material for the composites with the highest filler loading. Moreover, the higher particle agglomeration for samples with the highest filler loading is likely to increase filler–filler contacts which weaken the composite structure (Goonasekera et al., 2016; Grøndahl et al., 2017). For the P(3HO-co-3HD-co-3HDD) composite films, the sample with the lowest content of Se-Sr-HA showed the highest improvement of elastic modulus (14 MPa) and tensile strength (7 MPa) compared to neat P(3HO-co-3HD-co-3HDD) films (5.3 and 6.5 MPa, respectively). No increase in the elastic modulus was in fact evidenced for P(3HO-co-3HD-3HDD) samples containing 20 and 30 wt% of Se-Sr-HA compared to the ones with 10 wt% of filler and in parallel a reduction of the enthalpy of fusion (and consequently crystallinity) was obtained with the materials with 20 and 30 wt% of HA. The difference between the two types of PHAs might be related to the higher crystallinity of the scl-PHAs compared to the mcl ones. It has in fact been hypothesized that in polymers with high crystallinity, low quantities of filler present in the amorphous region do not impact significantly on the final bulk mechanical properties, therefore requiring higher filler amount compared to less crystalline materials (Kaur and Shofner, 2009; Grøndahl et al., 2017).

Overall, the P(3HB) composite containing Se-Sr-HA showed mechanical features similar to those of cancellous bone, making them good candidates for the development of materials for the reconstruction of defects of such type of tissue (Fernandez de Grado et al., 2018). However, future work should still be conducted to improve the brittleness of these materials, which might limit their *in vivo* application. On the other hand, P(3HO-co-3HD-3HDD) still showed a ductile behavior after the incorporation of the HA. Even though a drastic decrease in the deformation capability of the films was evidenced for the three filler contents analyzed, the composite samples with 30 wt% of Se-Sr-HA still showed a high elongation at break with an average value of 170%. Such a characteristic could make them a suitable candidate for the generation of scaffolds for non-load bearing applications, where their elastic nature might be an advantage for the development of flexible and easily shaped materials able to adapt to the defect site (Ural et al., 2000; Chen et al., 2012). Nevertheless, the final mechanical properties should be further optimized and could be achieved by improving the interface between the polymers and the filler with the use, for example, of nano-sized HA (Misra et al., 2008; Rai et al., 2008).

The main reason for the use of a co-substituted HA compared to traditional calcium phosphates was to develop a material possessing antibacterial properties to be used for the fabrication of the scaffold able to reduce and prevent the attachment and proliferation of bacteria in bone regeneration. The 2D composite films developed exhibited an antibacterial activity by two mechanisms, by direct contact between the bacteria and the selenite ions present in the HA structure (analyzed through the direct tests ISO 22196) and by action of the ions released in the surrounding environment (analyzed through the indirect contact test and antibacterial ions release). In particular, both composite types exhibited a high activity on contact, inducing at least a 90% reduction of bacterial cell number of both *S. aureus* 6538P and *E. coli* 8739. These results confirmed the activity of selenium containing compound against both Gram-positive and Gram-negative species, as already evidenced in MIC studies (Rodríguez-Valencia et al., 2013; Alam et al., 2016; Uskokovic et al., 2017). *S. aureus* 6538P and *E. coli* 8739 were chosen as representative organisms of Gram-positive and Gram-negative bacteria, respectively, as described by the ISO 22196. Future work will evaluate the antibacterial activity of the materials against other strains (e.g., *Staphylococcus epidermidis*, *Streptococci* sp., and *Pseudomonas* sp.) associated with bone infection and antibiotic-resistant strains such as methicillin-resistant *S. aureus.*

P(3HB) composites showed a higher effect compared to the P(3HO-co-3HD-co-3HDD) ones, as they completely prevented the growth of both Gram-positive and Gram-negative cells. This higher activity might be due to the differences in the surface properties of the two types of composites that were evidenced in the SEM analyses. The scl-PHAs samples possessed in fact a more porous surface which might have led to a higher exposure of HA on surface of the films, therefore potentially increasing the amount of ions in contact with the bacterial cells.

Both P(3HB) and P(3HO-co-3HD-co-3HDD) composites showed a reduction in the growth of *S. aureus* 6538P and *E. coli* 8739 during the *in vitro* antibacterial indirect tests. Such results can be used as an indication of the capability of such materials to release ions. In particular, the release of the ions seemed to possess an early onset as a reduction of the number of bacteria could be detected with the eluates obtained after 1 h of incubation for both types of PHAs. Moreover, it appears that the release is extended over time, as the media obtained at 24 h of incubation still possessed an antimicrobial activity. As expected, the materials containing a higher percentage of HA showed a higher antimicrobial effect which could be associated with an increased amount of selenite ions. Moreover, P(3HB)-based composites exhibited a slightly higher activity against both bacterial strains compared with composites with mcl-PHA matrix polymer. The higher porosity of P(3HB) matrix might be the reason of the higher antibacterial activity due to more rapid release of antimicrobial ions. Nevertheless, further studies should be conducted to quantify the concentration of the ions released and to investigate release kinetics over a longer period of time. For both P(3HB) and P(3HO-co-3HD-co-3HDD) composites, the growth inhibition was slightly more effective against Gram-positive bacteria than negative ones. In a few studies in the literature, low concentrations of selenite ions did not induce significant reduction in *E. coli* cultures (Vasić et al., 2011; Alam et al., 2016). It is known in the literature that some species are capable of reducing inorganic selenite ions to elemental selenium nanoparticles which are then deposited within the cell (e.g., in the cytoplasm or in the periplasmic space) or pumped outside the cells (Turner et al., 1998; Gonzalez-Gil et al., 2016). *E. coli* has been shown to possess such features probably making them less susceptible to the antimicrobial activity of such ions at lower concentrations (Turner et al., 1998). These results are also in agreement with the slightly higher MIC value obtained for *E. coli* 8739 compared to the *S. aureus* 6538P for the Se-Sr-HA.

## Conclusion

This work focused on tackling the worldwide threat posed by the rapid growth of antimicrobial resistance to antibiotics. In particular, we targeted bone regeneration, a biomedical application characterized by a high risk of bacterial infection which can significantly impair the correct regrowth of functional tissue. We reported, for the first time, the synthesis of a novel dual substituted HA containing selenium and strontium and its combination with two types of PHAs, both scl and mcl, to produce novel antibacterial materials for bone regeneration without the use of antibiotics. The loading of the filler in the polymer matrix induced an enhancement in the mechanical strength of both scl and mcl-PHA films, combined with a parallel reduction in the deformability of the materials. The developed composite materials possess high antibacterial activity against *S. aureus* 6538P and *E. coli* 8739 through two mechanisms, by direct contact between the bacteria and the materials surface and through the release of active ions at a concentration capable of inhibiting bacterial growth in a prolonged manner, up to 24 h. This study demonstrates the potential of these materials to provide antibacterial activity without the use of antibiotics, allowing them to be used as starting materials for the development of unique and effective antibacterial scaffolds for bone regeneration. Future work will focus on the development of these scaffolds and investigation of their biocompatibility properties using bone cell lines and further *in vivo* work.

## Data Availability Statement

The raw data supporting the conclusions of this article will be made available by the authors, without undue reservation.

## Author Contributions

EM produced the PHAs, performed all the experiments and data analysis related to the composite samples, and drafted the manuscript. MM produced the Se-Sr-HA, conducted the experiments and data analysis related to the Se-Sr-HA physiochemical characterization, and contributed to the drafting of the manuscript. RN contributed to the data analysis related to the characterization of the composite materials and the manuscript revision. MC, PJ, and PB contributed to the manuscript revision. JK was involved in the SEM/EDX analyses. AB and IR contributed to the planning and supervision of the project and manuscript revision. All authors contributed to manuscript revision and read and approved the submitted version.

## Conflict of Interest

MM is currently employed by company CAM Bioceramics B.V. MC and PJ are employed by Lucideon Ltd. During this research MM was however affiliated as a Ph.D. student at the University of Erlangen-Nuremberg, Germany and Lucideon Ltd. The remaining authors declare that the research was conducted in the absence of any commercial or financial relationships that could be construed as a potential conflict of interest.
